# Evaluation of the Use of Preventive Antibiotic Therapy in Patients Undergoing One-Step Prosthetic Revision Surgery with Low Preoperative Infectious Risk

**DOI:** 10.3390/antibiotics14030224

**Published:** 2025-02-21

**Authors:** Leonardo Motta, Giacomo Stroffolini, Stefania Marocco, Giulia Bertoli, Gianluca Piovan, Lorenzo Povegliano, Claudio Zorzi, Federico Gobbi

**Affiliations:** 1 Department of Infectious-Tropical Diseases and Microbiology, IRCCS Sacro Cuore Don Calabria Hospital, Negrar, 37024 Verona, Italy; leonardo.motta@sacrocuore.it (L.M.); stefania.marocco@sacrocuore.it (S.M.); giulia.bertoli@sacrocuore.it (G.B.); federico.gobbi@sacrocuore.it (F.G.); 2 Department of Orthopaedic and Traumatology, S. Cuore-Don Calabria Hospital, Negrar, 37024 Verona, Italy; gianluca.piovan@sacrocuore.it (G.P.); lorenzo.povegliano@sacrocuore.it (L.P.); claudio.zorzi@sacrocuore.it (C.Z.)

**Keywords:** prosthetic joint infection, early empiric antibiotic therapy, preventive antibiotic therapy, prosthetic knee infection, antibiotic stewardship, extended prophylaxis

## Abstract

**Introduction:** The prosthetic knee infection (PKI) rate in most centers ranges from 0.5 to 2% for knee replacements, depending on risk factors. Current PKI definitions may miss the identification of both early and late complications. There is no consensus on preventive or early empiric antibiotic therapy (EEAT) in the one-step exchange strategy for low-risk patients pending microbiology results. The aim of the study was to evaluate the potential role of EEAT in patients with comorbidities in preventing PKI and to evaluate differences in septic failure at 3, 6 and 9 months after prosthetic revision between patients undergoing EEAT and patients not undergoing EEAT. **Methods:** All adult patients undergoing one-step knee revision surgery at IRCCS Sacro-Cuore Don Calabria Negrar, from January 2018 to February 2021, were retrospectively included in a cohort observational study. Patients on antibiotic therapy before surgery or with preoperative ascertained PKI were excluded. Demographic characteristics, Charlson score, comorbidities, inflammatory markers, microbiological tests, imaging, infectious disease risk score and EEAT data were collected. Any postoperative complication or modification of antibiotic therapy at 14, 30, 90, 180 and 270 days after surgery was collected. **Results:** A total of 227 patients were included: 114 comorbid low-risk patients received EEAT after surgery, pending microbiological results; while 113 non-comorbid low-risk patients did not receive any antibiotic therapy in the postoperative period. Among the EEAT group, 16 were diagnosed with PKI, compared with 10 in the untreated group. Regarding septic failure during the 9-month follow-up after revision surgery, we registered nine cases in the EEAT arm and four in the untreated arm. In three out of nine cases treated with EEAT who had a post-revision septic failure, the causative microorganism was not successfully empirically targeted by EEAT; in the untreated group, two out of four cases had a post-revision septic failure, despite the targeted treatment of intraoperatively identified causative microorganisms. **Conclusions:** According to our results, EEAT after revision surgery in patients with comorbidities, who are at higher risk of infection, did not prevent prosthetic knee infections. There was also no evidence of a reduction in subsequent septic failure within nine months of revision surgery between groups. More accurate risk-defining scores are needed to identify patients at risk of PKI complications.

## 1. Introduction

Prosthetic joint infection (PJI) rates in most centers range from 0.5 to 2% for knee replacements, 0.5 to 1% for hip replacement and <1% for shoulder replacement [1,2]. In general, the PJI rate is highest during the first two years after primary implantation [3]. In a study involving more than 69,000 patients who underwent elective knee revision followed longitudinally from 1997 to 2006, the rate of PJI during the first two years after surgery was 1.5%; the rate of PJI 2 to 10 years after joint replacement was 0.5% [4]. In about 30% of the interventions that are proposed for revision, the cause is infective in origin.

According to current classifications, PJIs are defined based on the timing of surgery and the onset of symptoms, categorized as acute onset, early or delayed depending on the time of surgery, as well as chronic onset. However, these definitions are controversial and not always clear-cut, particularly in patients for whom diagnosis is chronic and delayed [5]. The main risk factors for PJI include the presence of comorbidities (rheumatoid arthritis, diabetes mellitus, malignant tumors, chronic kidney disease, obesity, lymphedema, immunosuppression), chronic use of immunosuppressive drugs, previous arthroplasty or previous surgical site infection, ASA score ≥3, prolonged duration of surgery, postoperative complications (hematoma, wound dehiscence) and bacteremia [1,2,3,4,5].

Early infection is usually contracted during implantation; less commonly, it may arise as a consequence of postoperative wound dehiscence with the contiguous spread of microorganisms from the skin to the joint cavity. Most early infections present with worsening joint pain, warmth, erythema, induration or edema at the incision site, wound drainage or dehiscence, joint effusion and fever. Delayed-onset infection is also often contracted during implantation; however, it typically presents with an indolent course, characterized by joint pain with or without loosening of the implant. Fever is present in less than half of the cases and there may be the presence of a fistulous passage. Delayed-onset infections are therefore difficult to distinguish from aseptic failure of the prosthetic joint.

Microbiological cultures may be negative in as many as 7–39% of patients with suspected PJI [6]. In general, cultures are more commonly positive in the case of early-onset PJI, but have a higher rate of negativity in patients with an indolent presentation in whom a prosthesis infection is not suspected and consequently insufficient or inadequate microbiological material is sent for analysis. Furthermore, the administration of antibiotics before surgery can cause false negative samples [3,7]. Additionally, there are other rare pathogens that can be a consequence of negative intraoperative cultures [8].

From a microbiological point of view, the biofilm plays a fundamental role in the context of prosthetic infections, and the microenvironment within the biofilm negatively affects the penetration and mechanism of action of antibiotics [9,10].

The diagnosis of PJI can be complex because definitions are not always agreed upon; several diagnostic criteria have been proposed, including the criteria of the European Bone and Joint Infection Society (EBJIS) in 2021 [11], the criteria of the 2018 International Consensus Meeting (ICM 2018) [12,13], the guidelines of the Infectious Disease Society of America in 2013 [14], and the modified criteria of the Musculoskeletal Infection Society (MSIS) in 2011 [15].

In general, the diagnosis is based on a combination of data, including medical history and physical examination, analysis of synovial fluid, markers of inflammation, culture data, radiology and intraoperative results [9]. According to these criteria, it is recognized that the diagnosis of PJI can be established in any of the following circumstances [11]: presence of a fistula communicating with the prosthesis; two or more intraoperative cultures positive for phenotypically identical organisms (≥2 intraoperative cultures or a combination of preoperative synovial fluid aspiration culture and intraoperative material culture); a single positive culture with a virulent organism (e.g., *S. aureus*, Gram-negative bacteria or streptococci) may represent a PJI. A single culture due to a relatively low-virulence organism (such as CoNS, *Corynebacterium* sp., *Cutibacterium* sp. or *Bacillus* sp.) is generally considered as a contaminant. Such organisms may be considered real pathogens if the same organism is found in at least two microbiological samples; in these cases, it is advisable to confirm the diagnostic suspicion of infection through other investigations, including intraoperative histology, which, however, is not always specific for infection and should be interpreted taking into consideration clinical history and alterations secondary to metallic debris [16]. Although a secondary criterion in PJI diagnosis, histopathological analysis remains relevant and should be assessed when available, including intraoperative frozen sections.

For cases where a definite diagnosis of PJI is not possible to agree upon, additional laboratory data (including serum inflammatory markers and synovial fluid examination) might be additional supporting elements for estimating the likelihood of infection. Routine serum inflammatory markers include erythrocyte sedimentation rate (ESR) and C-reactive protein (CRP) [9,17]. Other less commonly used serum markers are D-dimer, procalcitonin and interleukin-6. Routine synovial fluid analysis consists of cell count, Gram staining, aerobic and anaerobic culture and crystal analysis [9,17]. Other markers such as alpha-defensin and leukocyte esterase are not used routinely and do not have high diagnostic sensitivity and specificity, while D-lactate has been showing promising data [18].

The interpretation of the chemical–physical analysis of synovial fluid also depends on the timing of clinical presentation. In case of early-onset PJI, the synovial fluid cell count is diagnostic if it is >10,000 cells/µL (>90% neutrophils). In cases of delayed and late-onset PJI, the synovial fluid cell count is diagnostic if it is >3000 cells/µL (80% neutrophils).

Radiographic imaging can be useful but usually does not provide a definitive diagnosis of PJI. Conventional radiographs are useful for screening prosthesis mobilization and fractures but lack sensitivity and specificity for the diagnosis of PJI [9,19]. The radiological findings most commonly identified in the context of PJI are areas of osteolysis greater than 2 mm at the bone–cement interface, mobilization in the prosthetic component position, periprosthetic fractures and periosteal reaction [19,20]. However, these findings are not specific to infection but are also frequently observed in aseptic issues. Further second-level radiological investigations such as labeled leukocyte scintigraphy, positron emission tomography (PET), computed tomography (CT) and magnetic resonance imaging (MRI) are not used for routine diagnostic evaluation in most cases of suspected PJI but have a specific place in diagnostic workflow [19,20].

Scintigraphy (with technetium-labeled scans or gallium-67-labeled white blood cell scans), as well as PET-CT, are not useful in early infections because the scans may be falsely abnormal up to 12 months after surgery due to periprosthetic bone remodeling, and it has shown varying degrees of usefulness [21]. Furthermore, technetium-labeled scans can be positive in cases of aseptic loosening. A normal scintigraphy study can be considered evidence against the presence of infection (high sensitivity), but uptake, and therefore positivity, is not definitive for establishing the diagnosis of PJI (low specificity). There are CT techniques such as dual-energy or MRI MARS (metal artifact reduction system) that can be used and help to reduce metallic artifacts related to prosthetic implants, or that might be useful in diagnosing native osteomyelitis [19,20,21]. Some of the data that are useful in defining PJI according to shared guidelines are only available after surgery and therefore cannot be considered in the preoperative evaluation. In most cases, preoperative joint aspiration is not available due to a lack of joint fluid or the associated risk of the procedure itself. When considering the Philadelphia consensus [12,13], in patients who do not have a definite diagnosis of infection, an infectious risk score is assigned based on the combination of variables, and a score is calculated: if the score is ≤3, the prosthesis should be considered as non-infected, if ≥6, it should be considered infected. A case is defined as indeterminate when the score is between 4 and 5. When a PJI is confirmed (major criteria or score ≥ 6), the medical–surgical approach with the highest success rate involves the replacement of the prosthesis with an antibiotic spacer and subsequent reimplantation of a new prosthesis after a variable period (two-stage procedure). Although it is known that conditions such as diabetes, obesity, malnutrition, chronic kidney disease, COPD, rheumatoid arthritis and autoimmune diseases [22] increase the likelihood of infectious risk and, therefore, potential subclinical prosthesis infection (risk score between 4 and 5), the Philadelphia consensus score does not mention them, and it is unknown how much they may influence the preoperative evaluation.

Therefore, these patients may have an infectious risk score < 6 and may be classified as non-infected or indeterminate preoperatively, and may receive a diagnosis of PJI after intraoperative results with a subsequent high risk of failure.

In general, in the most updated guidelines there are no recommendations regarding the use of early antibiotic therapy in the postoperative care of patients undergoing revision joint replacement surgery if not scored at risk. However, in the literature, some studies suggest the use of postoperative antibiotic therapy in selected high-risk patients after revision surgery to reduce the risk of septic recurrences or new infections in the months following the intervention. Particularly, in 2018, Inabathula et al. published the results of a retrospective study that examined the rates of PJI at a single center before and after the implementation of postoperative oral antibiotic therapy for high-risk total hip arthroplasty (THA) and high-risk total knee arthroplasty (TKA) [23]. The infection rates at 90 days were 1.0% and 2.2% for TKAs and THAs, respectively. High-risk patients without prolonged antibiotic prophylaxis were 4.9 times (*p* = 0.009) and 4.0 times (*p* = 0.037) more likely to develop PJI after THA and TKA, respectively, compared to high-risk patients with extended antibiotic prophylaxis. This study did not demonstrate statistical significance for adverse effects associated with the therapy in both subgroups. Another study by Claret et al. in 2015, involving 341 patients undergoing aseptic knee prosthesis revision, showed a lower PJI rate in patients receiving postoperative antibiotics with teicoplanin and ceftazidime for 5 days compared to untreated patients (2.2% versus 6.9%, *p* = 0.049) [24]. Another study by Kuo et al. in 2019, involving 418 patients undergoing aseptic hip revision surgery, demonstrated that the incidence of PJI was 4.8% in patients who received extended antibiotic prophylaxis compared to 2.4% in patients who received standard prophylaxis, with no statistically significant difference [25].

In summary, current PKI definitions and criteria may miss the identification of both of chronic PJI. There is no consensus over EEAT, extended prophylaxis or preventive therapy in the one-step exchange strategy for patients pending microbiology results [26]. The aims of the study were to evaluate the potential role of EEAT in patients with comorbidities in the early treatment of preoperatively undiagnosed PJI and to prevent in high-risk patients a new-onset PJI by evaluating the difference in septic failure at 3, 6 and 9 months after prosthetic revision between patients undergoing EEAT and patients not undergoing EEAT.

## 2. Results

### 2.1. Diagnosis of PJI at 14 Days Post-Surgery

A total of 227 patients were included, of whom 114 (50.2%) received EEAT, while 113 (49.8%) did not. In the EEAT group, 98 patients (85%) were not diagnosed with PJI in the postoperative period, indicating overtreatment, while 16 (15%) reached a score of six or higher due to microbiological isolates, resulting in a diagnosis of PJI. Adverse effects events were registered for six (5.3%) of patients in the EEAT group and none in the other group (see Table 1). The difference between the two groups was not statistically significant (χ^2^ = 1.0368, *p* = 0.30).

Among these diagnosed 16 cases, the most frequently identified pathogens were *Coagulase-negative staphylococci* (CoNS) in 14 cases (87%) and *Staphylococcus aureus* in 2 cases (13%). These were susceptible to EEAT. In the non-EEAT group, 103 patients (91%) had either negative or non-diagnostic microbiological results for PJI, while 10 patients (9%) were diagnosed with PJI and continued treatment. Among these cases, *Coagulase-negative staphylococci* (CoNS) were identified in 70% (n = 7), *Cutibacterium acnes* in 10% (n = 1), Gram-negative bacteria in 10% (n = 1) and streptococci in 10% (n = 1).

Summing up, the diagnosis of PJI was not suspected preoperatively in a total of 26 cases (11.45%).

Out of these 26 patients, only in 4 cases was a preoperative joint aspiration performed, and none of these exams isolated a pathogen, nor did the synovial fluid show >3000 leukocytes/mm^3^, possibly indicating PJI at the preoperative evaluation.

Out of the 26 patients, bone scintigraphy with labeled leukocytes was performed in 5 cases, but it showed non-specific inflammation that was inconclusive for determining PJI.

In all 26 patients, a preoperative dual-energy knee CT scan was performed to reduce metallic artifacts and provide better visualization of periprosthetic soft tissues. However, there were no particularly suggestive elements of PJI. Among the most common findings worth noting were soft tissue calcifications (6/26), periprosthetic osteolysis areas (3/26) and mobilization (3/26), all of which were considered aseptic in all cases (see Table 2). No differences between different risk scores were found.

### 2.2. Septic Failure (Post-Surgical)

A total of nine patients in the group that received EEAT and four patients in the group that did not underwent a new revision due to septic failure (post-surgical). The difference between the two groups was not statistically significant (χ^2^ = 1.2684, *p* = 0.26).

In the first group, out of these nine patients, four received a diagnosis within 28 days of the intervention and underwent a DAIR or m-DAIR procedure, while five received a later diagnosis within 9 months of the intervention (see Table 3).

Out of these, three had microbiological positivity intraoperatively at the time of reimplantation, confirmed within 14 days. It is noteworthy that all patients from this group with a microbiological confirmed infection underwent DAIR (3/3).

For those patients in the EEAT group, the most frequently identified comorbidities were diabetes mellitus, present in 77% of cases, rheumatological diseases (rheumatoid arthritis, psoriatic arthritis or connective tissue diseases) in 33% and hematological diseases in 11%.

The bacteria involved included CoNS in five cases and *S. aureus* in two cases, while in the remaining cases, a microbiological diagnosis was not reached, but there was evidence of an intraoperative appearance of purulence.

In the group of patients not undergoing EEAT, there were four cases of septic failure within 9 months. In one case, a DAIR procedure was performed (acute onset), while in the remaining three cases, the diagnosis was made within 9 months following the surgical intervention.

The bacteria involved were *S. lugdunensis* (1/4), *P. aeruginosa* (1/4) and CoNS (2/4).

### 2.3. CoNS Resistance Patterns

Since our center has observed infections caused by CoNS (identified in multiple samples), we conducted a microbiological analysis of isolates from orthopedic cases to determine the most effective EEAT strategy. Among the isolates from patients who underwent one-step knee revisions between 2018 and February 2021, a total of 97 CoNS strains were identified. The most frequent among these was *S. epidermidis* (46, 47.4%), followed by *S. hominis* (29, 29.8%), *S. warnerii*, (9, 9.27%), *S. lugdunensis* (8, 8.2%), *S. capitis* (3, 3.1%) and *S. sciurii* and *S. xylosus* (1.03%, 1, 1.03% each). Among all these microbiological isolates, the percentage of methicillin resistance was 31% (30 strains), and the presence of resistance (MIC greater than or equal to 4) to teicoplanin was 17.5% (17), with 2 of these maintaining susceptibility to methicillin and exhibiting an isolated profile of altered susceptibility to glycopeptides (see Table 4).

## 3. Discussion

This investigation was undertaken to comprehensively analyze the recent collaborative efforts between the Infectious Diseases Service and the Orthopedics Service at Negrar Hospital, examining both microbiological and clinical aspects. The primary objective was to assess the optimization of preoperative management for patients undergoing one-step prosthetic revision surgery.

The study findings indicate that, while distinguishing between two distinct populations—patients with comorbidities possibly predisposing them to prosthetic infection and those without such comorbidities (high/low risk)—no statistically significant difference was observed (χ^2^ = 1.0368). Although the sample size was limited, the group receiving EEAT demonstrated a higher absolute number of infections that were not identified preoperatively. Additionally, there were more cases of septic failures within nine months post-reimplantation in this group (6 vs. 2, not statistically significant). A larger patient cohort might have provided greater clarity regarding potential statistically significant differences. It would be of great interest to determine a further scoring system that could actually identify which of these patient may benefit for an additional evaluation for estimating the risk of septic failures, as recent experiences have described [27]. New informatic and technological tools may serve this end.

PJI was overall not suspected in 26 cases (11.45%). We investigated whether these cases, clinically and through laboratory techniques not suggestive of infection, could have been predicted through imaging techniques or preoperative joint aspiration. Only four of these cases underwent preoperative joint aspiration, yielding no pathogens or synovial fluid with >3000 leukocytes/mm^3^, and other patients had a “*puntio sicca*” or no synovial fluid to obtain. Imaging techniques showed low sensitivity and specificity, with inconclusive radiological findings.

Diagnosing prosthetic infection preoperatively in the absence of clear clinical signs is challenging.

The standardization and implementation of preoperative diagnostics for patients with even slight clinical suspicion of PJI are essential. Arthrocentesis may not always be feasible, and imaging, especially dual-energy CT, could be useful, particularly in the presence of calcifications in periarticular soft tissues. Intraoperative findings, surgeon experience, symptom onset timing and medical history contribute to decision-making, with an emphasis on obtaining samples as aseptically as possible to prevent false positives. In our cohort, we could not assess the value of applying sonication, syndromic multiplex or targeted PCR and next-generation sequencing, which would have possibly added significant information [28,29]. Moreover, without a sequencing targeted program, it is harder to follow the evolutionary trajectories of *staphylococci*, which have been suspected of being possibly clonal in our institution due to their resistance profile [30]. More tools need to be implemented in our setting and across hospitals in order to provide additional microbiological detection capability.

Prolonged antibiotic therapy did not reduce new septic failures and resulted in overtreatment in 98 out of 114 cases. However, among the 16 cases not identified preoperatively, 12 (70%) were successfully managed with EEAT, highlighting its potential effectiveness in treating infections. In the same group, 4 cases required subsequent revision surgeries. Similarly, in the non-EEAT group, 10 patients had infections diagnosed intraoperatively based on microbiological findings and received targeted therapy. Of these, 8 (80%) achieved successful infection management, preserving the prosthesis without further intervention.

These combined results demonstrate the overall effectiveness of a combined multidisciplinary approach in managing infected PJI, even in cases not identified preoperatively, and possibly with a one-step approach in selected cases. This is in line with other data from other centers, and should possibly become a standard to ensure better outcomes across institutions [31]. As a note, no statistically significant differences were observed between the EEAT and non-EEAT approaches regarding outcomes, although a few adverse events were noted in the EEAT group.

Concerning therapy, in our institution, broad-spectrum antibiotic use has possibly led to resistance development, notably against teicoplanin (17.5% resistance) which was used also in the EEAT protocol.

In summary, no major statistically significant differences were found pertaining to the outcomes in the two groups (EEAT, non-EEAT), in order to treat patients not identified preoperatively, and in consideration of antibiotic stewardship principles, it can be stated that it is safe not to apply the EEAT approach to these patients to avoid resistance development and AEs, still keeping the high success rate of one-step revisions. These results are in line with a recent major study in which no apparent association between the type or duration of antibiotic prophylaxis and the risk of complete revision for infection was found, adding evidence questioning whether there is any advantage to the use of prolonged antibiotic prophylaxis beyond a single dose [32]. One of the main limitations of this study is the sampling method and population size, which may impact the statistical power of our findings. While this approach is dictated by the retrospective design of the study and the specific type of infection, it may limit the generalizability of our results. Another limitation of our study is that current risk stratification criteria may not adequately distinguish between low-risk and intermediate-risk patients regarding the impact of EEAT. Despite adjustment analyses, no significant differences emerged, suggesting that comorbidities remain difficult to quantify in this context. Finally, we acknowledge that the absence of routine histopathological examination represents a limitation of the study, as it may impact the accuracy of diagnosing PJI and could lead to potential false positive or false negative culture results. Future prospective studies with predefined power analyses, namely larger, well-stratified cohorts and refined risk assessment models are needed to better address this issue. In particular, a randomized controlled trial (RCT) would be welcome for evaluating postoperative antibiotic therapy, given conflicting literature data.

## 4. Materials and Methods

This study reports data from a retrospective monocentric cohort study conducted at IRCCS Sacro Cuore-Don Calabria, which is a regional reference center for prosthesis revision. Eligible participants included all patients undergoing total knee arthroplasty revision between January 2018 and February 2021. All eligible patients signed informed consent, Institutional Review Board approval 30 September 2021 (Prog CE. 3441CESC).

Since January 2018, in our facility, following the approval of internal protocols between the Department of Orthopedics and the Department of Infectious, Tropical and Microbial Diseases (DITM), the preoperative assessment of all patients undergoing prosthesis revision has been multidisciplinary. The task of the infectious disease (ID) specialist is to evaluate the infectious risk, specifically based on the 2018 Philadelphia consensus [12,13,14,15], and in the case of confirmed prosthetic infection, according to guidelines, recommend a two-stage revision. If the risk score is intermediate or low, the treatment plan is assessed on a case-by-case basis in agreement with the orthopedic surgeon and following international guidelines. At our center, patients with an infectious risk score between 3 and 5 and at least one condition among diabetes, obesity, malnutrition, chronic kidney disease, COPD, rheumatoid arthritis and autoimmune diseases are recommended for EEAT. For a one-step prosthesis revision, patients are prescribed early empiric broad-spectrum antibiotic therapy (EEAT) targeting both Gram-positive and Gram-negative bacteria. The standard regimen includes teicoplanin at 10 mg/kg/day (following a loading dose) and ceftriaxone 2 g/day, provided there are no known drug allergies and renal function is normal. This may be considered a sort of extended prophylaxis or preventive therapy pending microbiology results. During the procedure, 5 to 7 intraoperative samples from subcapsular and intracanal material are collected for microbiological analysis, with final results available within 14 days. It is the institution’s protocol to send all revision cases for microbiological analysis, even in the absence of clinical or operative suspicion of PJI. In cases of allergy to third-generation cephalosporins, teicoplanin is paired with ciprofloxacin; for teicoplanin allergy, linezolid is used as an alternative. On the other hand, EEAT is not prescribed for patients when the aforementioned comorbidities are absent. The objective of this therapeutic antibiotic intervention is twofold: firstly, to treat any PJI that is not suspected during preoperative evaluation and, secondly, to minimize the postoperative infectious risk. Within two weeks from the procedure, the ID specialist decides whether to continue or suspend antibiotic therapy based on all intraoperative microbiology results. Patients diagnosed with a PJI according to the international guidelines (2018 consensus [12,13,14,15]) are then re-evaluated in a multidisciplinary team and followed up.

**Figure 1:**
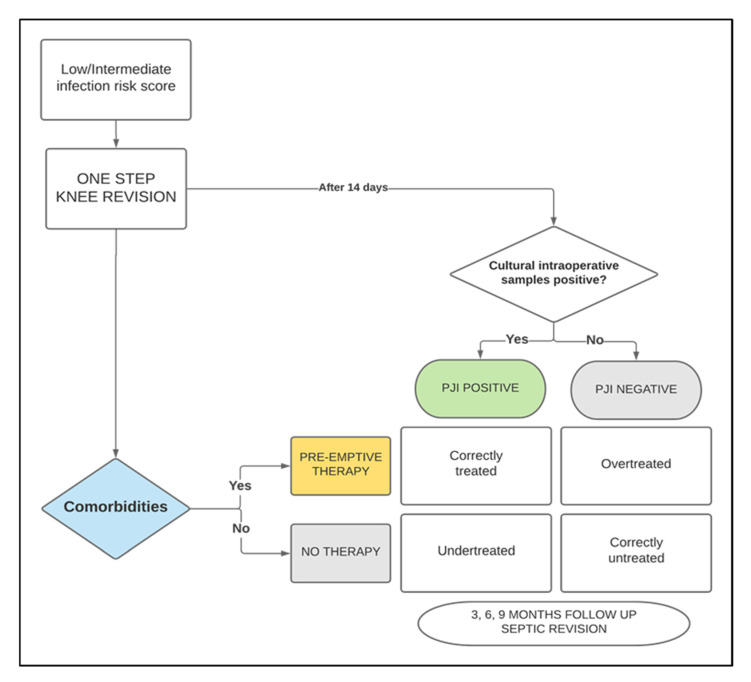
Flowchart of the study.

Flowchart of the study is shown in Figure 1.

For the purpose of this study, the demographic characteristics, Charlson score, inflammatory markers (PCR, ESR, D-dimer), any microbiological isolate, imaging report, and infectious risk score regarding the preoperative evaluation are reported; the characteristics of early empiric antibiotic therapy (EEAT) initiated at time 0 (after surgery) are reported. Inflammatory markers, any microbiological isolate (including antibiograms), postoperative complications (fever, surgical wound complications) and any modification/suspension of therapy at 14 days post-surgery are documented; inflammatory markers, any microbiological isolates with their respective antibiograms, postoperative complications (fever persistence, surgical wound complications) and any modification/suspension of antibiotic therapy at 30, 60 and 90 days post-surgery or until the onset of infectious complications (within 9 months after the revision surgery) are recorded.

The primary objective of this study was to evaluate whether our approach of early empiric antibiotic therapy in patients with low infectious risk but comorbidities results in overtreatment based on the definitive diagnosis of PJI obtained from intraoperative microbiological tests at 14 days after revision surgery.

Therefore, the primary endpoint was the diagnosis of PJI in accordance with the 2018 Philadelphia and 2013 Musculoskeletal Infection Society (MSIS) criteria at 14 days following the revision surgery.

The secondary objectives were to describe the occurrence of septic failure and orthopedic revision in patients undergoing one-step revision without a subsequent diagnosis of PJI (negative intraoperative samples) at 3, 6 and 9 months after the revision surgery and assess the percentage of resistance to glycopeptides and methicillin resistance in the microbiological isolates.

Inclusion/Exclusion Criteria:

Inclusion Criteria:

- Adult patients undergoing one-step PTG revision surgery between January 2018 and February 2021.

Exclusion Criteria:

- Patients receiving antibiotic therapy before surgical intervention.
- Patients with confirmed prosthetic joint infection.
- Patients with a history of previous PJI.

### 4.1. Statistical Analysis

#### 4.1.1. Sample Size Calculation

Given the retrospective nature of the study, a formal sample size calculation was not conducted. Data were collected from eligible patient medical records between January 2018 and February 2021. Considering that in our center there are approximately 100 patients undergoing one-step PTG revision surgery each year, it was estimated that around 300 patients would be included.

#### 4.1.2. Planned Analyses

Demographic and clinical patient characteristics are summarized using descriptive statistics. Continuous variables are summarized using descriptive statistics, including the number of observations, mean and standard deviation or the minimum, maximum and median. Categorical variables are summarized using absolute and relative frequencies. The presence of any missing data will be appropriately reported, indicating the percentage of valid cases relative to the total. Two-tailed tests with a significance level of 5% will be used unless otherwise specified. When and if appropriate, the outcomes of interest are reported with their respective 95% confidence intervals. Analyses have been performed using SAS EG 7.1 statistical software. The number of patients with a definitive diagnosis of PJI and the number of subjects undergoing EEAT are reported in a contingency table from which absolute and relative frequencies of the subgroups of interest can be calculated. The frequency of overtreatment has been defined by the number of subjects undergoing EEAT divided by the number of subjects without PJI (1-Sp), while the frequency of undertreatment is defined by the number of subjects not undergoing EEAT among those testing positive for PJI (1-Se). The proportion of microbiological isolates susceptible to therapy are reported through absolute and relative frequencies. The Charlson score and the proportion of patients with negative intraoperative samples who experienced septic revision at 3, 6 and 9 months are also described using absolute and relative frequencies.

## 5. Conclusions

This study highlights the importance of a multidisciplinary approach in identifying patients at risk of prosthetic joint infection (PJI), where appropriate risk stratification and targeted prophylaxis can help to prevent unnecessary prolonged antibiotic therapy. By ensuring that antibiotics are used when genuinely necessary, we can reduce the risk of patient safety issues and prevent the emergence of resistant bacterial strains. The daily challenges addressed in this study stem from the difficulty of managing complex implant-related infections, while striving to prevent septic complications. This reinforces the need for collaborative efforts with orthopedic colleagues to optimize antibiotic therapy, aligning with the principles of antimicrobial stewardship to balance effective infection prevention and the prudent use of antibiotics.

## Figures and Tables

**Table 1:** Postoperative outcomes of EEAT or No-EEAT.

	Low/Intermediate Risk Score Patients with Comorbidities (EEAT)	Low/Intermediate Risk Score Patients Without Comorbidities (No-EEAT)
**Unknown PJI**	16	10
**No PJI**	98	103
**AEs**	6	0

EEAT: early empiric antibiotic therapy; PJI: prosthetic joint infection; AEs: adverse events.

**Table 2:** Diagnostic characteristics of patients in which PJI was not suspected preoperatively and then diagnosed intraoperatively.

Synovial Analysis	Scintigraphy	Dual Energy CT Scan
No alterations (4/4)	Non-specific findings (3/26)	Soft tissues calcifications (6/26) Areas of osteolysis (3/26) Prosthesis mobilization (3/26)

CT: computed tomography.

**Table 3:** Total septic failures according to EEAT or No EEAT groups.

	Total Septic Failures	DAIR	1/2 Step Replacement
**EEAT**	9	4	5
**No EEAT**	4	1	3

DAIR and or 1/2 step replacement approach. EEAT: early empiric antibiotic therapy. DAIR: debridement, antibiotic and implant retention.

**Table 4:** Microbiological characteristics of the involved *Coagulase-negative staphylococci* (CoNS).

Total CoNS = 97
N. CoNS MS = 69 (69%)
N. CoNS MR = 30 (31%)
N. CoNS R to teicoplanin = 17 (17.5%)

MR: methicillin-resistant; MS: methicillin-susceptible; R: resistant.

## Data Availability

Data available on request due to restriction due to ethical, legal and privacy reasons.
